# Perceived Work Conditions and Turnover Intentions: The Mediating Role of Meaning of Work

**DOI:** 10.3389/fpsyg.2016.00704

**Published:** 2016-05-12

**Authors:** Caroline Arnoux-Nicolas, Laurent Sovet, Lin Lhotellier, Annamaria Di Fabio, Jean-Luc Bernaud

**Affiliations:** ^1^Centre de Recherche sur le Travail et le Développement, Conservatoire National des Arts et MétiersParis, France; ^2^Department of Education and Psychology (Psychology Section), University of FlorenceFlorence, Italy

**Keywords:** meaning of work, work conditions, turnover intentions, mediation, French workers

## Abstract

Perceived working conditions lead to various negative outcomes for employee behaviors, including turnover intentions. Although potential mediators for these relationships were previously identified, the importance of meaning of work has not yet been investigated. This study examines the role of this psychological resource as a mediator for the relationships between perceived working conditions and turnover intentions in a sample of 336 French workers from different job contexts. Results show that adverse working conditions were positively and significantly associated with turnover intentions. Meaning of work is negatively related to both perceived working conditions and turnover intentions. Mediation analyses for meaning of work demonstrated indirect effects of several adverse working conditions on turnover intentions. The role of meaning of work as a psychological resource for employees facing adverse working conditions is discussed, especially regarding its implications for research and practice within organizational contexts.

## Introduction

The fifth European survey on working conditions (Eurofound, 2012) conducted in 34 countries on a sample of 44,000 workers, showed the essential role of relation to work in the individuals’ lives. According to this study, exposure to physical risks in the workplace has not decreased since 1991 and psychosocial risks may have harmful health consequences in the current socioeconomic situation, which is characterized by high demands, work intensification, lack of autonomy, insufficient social ties, and a sense of insecurity toward work. Several studies have shown that the collateral effects of such work stress and psychosocial risks could have societal, organizational, sectoral, and individual costs (Hoel et al., 2001; European Agency for Safety and Health at Work, 2014). In particular, 20% of workers report a poor mental well-being and 18% of them a poor work-life balance. Moreover, the Eurofund study points out the limited changes in working conditions over the last 20 years and the fact that unfavorable working conditions tend to affect disproportionally some groups of workers.

Research has shown the links between poor working conditions and intentions to leave. Workers in hazardous workplace conditions are indeed more likely to leave their current employers voluntarily and in case the employer does not take the necessary measures in order to improve the work conditions, workers will not give up their withdrawal intentions (Cottini et al., 2011). As a result, the importance of the relation to work conditions cannot be underestimated. One of the benefits of this research is in labor relations, especially for organizations that attempt to retain some of their employees because, according to Bertrand et al. (2010, p. 214), companies must also “ensure talent retention” and “maintain and develop the expertise.” Indeed, a major challenge for organizations is the significant cost of staff turnover in terms of recruitment and training of personnel. The financial loss caused ranges from a few thousand to more than double the individual’s salary and the adverse effects on organizational performance and group motivation are significant (Singh and Loncar, 2010).

The present contribution considers the impact of working conditions on the intention to leave a job. It then successively examines the concepts of meaning of work. Assuming the influence of work conditions on turnover intentions, it then explores the consequences of working conditions on meaning of work that lead to suggest mediating effects of meaning of work on these two variables.

### Effects of Work Conditions on Turnover Intentions

Working conditions may have various positive and negative impacts on employees’ outcomes such as turnover intentions. Different research on various working samples have shown that perceived work conditions may affect turnover intentions (Houkes et al., 2001; Huang et al., 2007; Podsakoff et al., 2007; Poilpot-Rocaboy et al., 2011; Burakova et al., 2014). Mueller and Price (1990) have established that the determinants in voluntary turnover are of a psychological, sociological, and economic nature. Their explanatory model of voluntary turnover integrates different types of determinants, such us working conditions, environmental conditions, and employee characteristics. The authors point out that if employees’ expectations toward the organization are not fulfilled, the consequences for job satisfaction and commitment to work result in the employees deciding to leave the organization.

In this regard, Dawis and Lofquist (1984) argue in their model that the degree of satisfaction from the perspective of the employee as well as of the employer predicts the extent to which the individual is likely to stay. In case of a mismatch between the person and the working environment, this model predicts forms of adjustments between the two. Thus, active adjustment on the part of the individual implies that he or she is trying to change the working environment. Adjusting reactively, individuals may also change their behavior to better match the environment. When no more adjustment proves possible, the person leaves the job. According to Mobley et al.’s (1978) model, that explains the withdrawal process, cognitive behavioral variables are mediators of the relationship between satisfaction and employee’s turnover. This conceptual model describes the cognitive process in which job dissatisfaction leads the individual, at first, to think of leaving, and then to intend to leave, which is accompanied by the active search for another job, resulting in the decision to leave if an interesting job offer arises.

Mobley (1977) distinguishes the intention of seeking a new job and the intention to leave and says that the intention of seeking and the resulting job search generally precede the intention to leave and actual turnover except in cases of impulsive behavior. Work dissatisfaction is a factor that leads the individual to explore new alternatives (Peake and McDowall, 2012). Mobley et al. (1979) mention the negative relationship between turnover and the age, position, job content, intention to stay in the current position, commitment, and job satisfaction. They point out that less than 20% of the turnover variance is explained. Other explanatory factors have been identified. Mitchell et al. (2001) explained withdrawal intentions with new processes, adding factors that influence the decision to leave, such as satisfaction and commitment, the comparison between the current situation and the future situation, and the occurrence of particular life events. Several studies have confirmed the influence of job satisfaction and organizational commitment on withdrawal intentions (Cossette and Gosselin, 2009). Various authors have highlighted the moderate negative correlation between job satisfaction and turnover, as well as the negative relationship between commitment and turnover (Porter and Steers, 1973; Mobley et al., 1979). In his study based on a sample of maintenance technicians, Maghni (2014) shows that the variance of the withdrawal intention is explained by 34.7% work satisfaction (intrinsic and extrinsic) and 12.6% by organizational commitment.

While some studies have reported on the deleterious effect of adverse working conditions on health (Conne-Perréard et al., 2001), many authors have highlighted the protective role of certain psychological resources in facing difficult working conditions. For example, some authors (Marc et al., 2011) identified professional isolation as a psychosocial risk factor. In a study on psychosocial risk factors, Bué et al. (2008) emphasized the protective effect of social support when facing difficult working conditions while other authors (Caron and Guay, 2005) demonstrated the link between social support and mental health. If satisfaction is a mediator of withdrawal intentions, it would be relevant to analyze whether the meaning of work could act as a mediator between perceived work conditions and intentions to leave, which to our knowledge, has not yet been explored. None of the existing studies have considered meaning of work as a mediator variable.

### Conceptualizing Meaning of Work

The concept of meaning of work features a variety of definitions. It usually refers to a subjective experience that has a personal meaning for the individual (Rosso et al., 2010). Steger et al. (2012, p. 323) define the meaning of work not only as “all that work means for individuals” (*sense*) but also as having “significant and positive valence” (*meaning*). If it is a subjective and personal experience, sense refers primarily to the experience of coherence, cohesion, balance, or wholeness. According to Morin (2006), it corresponds to an experience of coherence and balance between the features that the individual seeks in the work and those he or she actually finds in the work. For Frankl (1969), the meaning of work is associated with the purpose and the reason for living as well as with the vocation.

One common idea is that the emergence of meaning requires the presence of a goal or a cause that transcends the life of the individual (Frankl, 1969). From the perspective of spirituality, Seligman (2003) expresses the idea that finding meaning leads to make a connection with a sphere that extends beyond us. Individuals transcend themselves; that is to say, go beyond themselves to find meanings that are distinct from themselves (Frankl, 1959). According to Ashmos and Duchon (2000), the expression of spirituality at work requires accepting the idea that employees want to be involved in work that gives meaning to their lives. In general, there is a link between the overall level of the meaning of life and of the meaning of work (Steger and Dik, 2009). On the whole, such meaning, through the direction and consistency it gives to the actions of the individual, thereby conveys a structuring framework.

### Meaning of Work as a Mediator

Some studies have highlighted the role of the meaning of life as a mediator in difficult life situations. Thus, in their study of adolescents from poor families, Machell et al. (2015) show the moderating role of the meaning of life on antisocial behavior such as disobedience and bullying. These authors consider the meaning of life as a structured framework for young people that enables them to resist anti-social behavior. Hence the idea arises that meaning could act as a framework or a protective firewall, especially in adverse situations.

By extension, one could consider the meaning of work as a firewall in the work context in terms of the structural framework, coherence, and objectives that meaning gives to work activities and that transcend the individual. Moreover, meaning of work is a more salient concept than meaning of life within an organizational context, and thus has a direct influence on employees’ behaviors and subjective experiences of work (Ardichvili, 2005; Rosso et al., 2010; Steger et al., 2012). To that extent, the job characteristics model (JCM) developed Hackman and Oldham (1976) suggested that meaning of work can act a mediator between job characteristics (variety of skills, tasks, characteristics, autonomy, and feedback) and the various employee’s outcomes such us motivation, satisfaction, and in particular, turnover intentions. This result was confirmed by the meta-analysis conducted by Humphrey et al. (2007). Indeed, they identified meaning of work as the most important mediator between working conditions and turnover intentions. Consequently, we wanted to reexamine these relationships among a sample of French workers.

## Purpose of the Study

**FIGURE 1:**
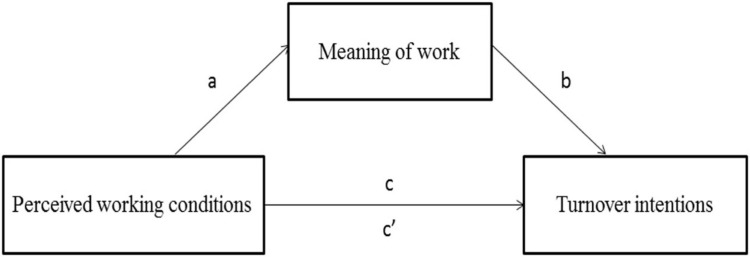
**Hypothetical mediating model of meaning of work in the relationships between perceived working conditions and turnover intentions**.

The present study extends prior research on meaningful work by exploring its potential mediating effects on the relationships between organizational context and employee outcomes following the JCM (Hackman and Oldham, 1976). More specifically, our focus was to examine how the relationships between working conditions and turnover intentions are mediated by meaning of work among a sample of French workers (see **Figure 1**). Consequently, four hypotheses were formulated:

Hypothesis 1: Adverse working conditions are positively associated with high turnover intentions (path *c*).
Hypotheses 2 and 3: Meaning of work is negatively related to adverse working conditions (path *a*) and high turnover intentions (path *b*).
Hypothesis 4: The effects of adverse working conditions on turnover intentions are partially mediated by meaning of work (path *c’*).

## Materials and Methods

### Participants

Participants were 336 employees from different organizations and institutions. The sample included 98 males and 238 females (*M*_age_ = 42.38, *SD*_age_ = 10.47). A large majority of respondents reported being engaged or married. In terms of education level, 43 respondents had the equivalent of a high school diploma or less (13%), 92 had attended or completed a bachelor’s degree program (27%), and 201 had attended or completed a graduate degree program (60%). Various occupations were represented but administrative or managerial positions were more common (60%). Social services (15%), public administration (14%), education (13%), and scientific and technological activities (7%) were the most occupations mentioned by respondents. Among the participants, 240 were full-time employees (71%), 90 were part-time employees (27%), and six did not report their contract status. Most of respondents were in permanent position (75%), 33 were in temporary position (10%), 32 were self-employed (9%), and 19 reported non-standard employment such as interim or State-aided contracts (6%). Their work experience ranged from less than 1 year to over 40 years, while mean job tenure in their current position was 7.09 years (*SD* = 7.01).

### Measures

#### Adverse Work Conditions

The Questionnaire of Adverse Work Conditions Experience (Bertrand et al., 2010) includes 45 items that measure employees’ experience with various adverse work conditions. Initially, the authors identified eight aspects of adverse work conditions among French-speaking Belgian workers by conducting exploratory factor analysis. These factors consist of work pressure (nine items, e.g., unforeseen workload), lack of resources (six items, e.g., lack of social support when needed), job insecurity (six items, e.g., unacceptable aspects of employment contract), organizational changes (seven items, e.g., changes in working conditions), lack of personal development (five items, e.g., routine job), personal reasons (five items, e.g., health problem), work climate (five items, e.g., hostile atmosphere among colleagues), and public image of the company (two items, e.g., poor public perception). The questionnaire prompts participants to respond “yes” or “no” as to whether or not they experience such a situation in their current job. Following the instructions provided by the original authors, eight adverse work conditions indexes were built by calculating the average number of “yes” responses. For instance, if a participant answered “yes” to six of the nine items from the work pressure dimension, an index of 0.67 was obtained. Kuder–Richardson coefficients of reliability or K–R 20 were unacceptable for job insecurity (K–R 20 = 0.40) and for work climate (K–R 20 = 0.42; Nunnally and Bernstein, 1994). These results can be explained by the low variation that was observed. Indeed, few employees experienced very adverse working conditions. Then, K–R 20 for the personal reasons dimension and for public image of the company were acceptable, with scores of 0.60 and 0.67, respectively. For the other factors, K–R 20 scores were good ranging from 0.73 (lack of resources) to 0.76 (lack of personal development). After excluding the two factors below 0.60, the K–R 20 coefficient for the total scale in this study was 0.85.

#### Turnover Intentions

The Questionnaire of Turnover Intentions (Bertrand et al., 2010) consists of five items that measure employee turnover intentions using a 4-point Likert scale ranging from 1 (*Strongly disagree*) to 4 (*Strongly agree*). One example item is: “I have the intention to leave my company.” The original authors also conducted an exploratory factor analysis among the same sample providing evidence of the unidimensionality of the scale. The Cronbach’s alpha was 0.84 in this study.

#### Meaning of Work

The Meaning of Work Inventory (Arnoux-Nicolas et al., 2016) is a 15-item scale that assesses meaning of work using a 7-point Likert scale ranging from 1 (*strongly disagree*) to 7 (*strongly agree*). It consists of four dimensions: importance of work (e.g., “My current job gives meaning to my life”), understanding of work (e.g., “I understand the value of my work”), direction of work (e.g., “My work has a clear and specific direction”), and purpose of work (e.g., “I frequently don’t understand the purpose of my work”). A higher-order factor was also found suggesting that the total score can be used as an overall measure for meaning of work. Since the mediating role of meaning of work was investigated, this global indicator was preferred for the current study. The Cronbach’s alpha was 0.90.

#### Procedure

In order to collect data among employees with different job status and from a range of organizations, participants were recruited in two ways: using our personal and professional networks; and, for employees located in the Paris region, by in-person contact. The former were directly contacted by e-mail, while the latter were given a flyer advertising our survey. In both cases, an electronic link was provided to access the online survey. All respondents were required to review legal information and provide their consent before participating in the survey. This research is not invasive, is anonymous and voluntary. Using a snowball sampling strategy, potential participants were encouraged to forward this link to their acquaintances. A similar approach was applied by Steger et al. (2013) to increase their pool of participants.

The project and ethical features of this research were approved by the Doctoral School Abbé Grégoire of the Conservatoire National des Arts et Métiers in Paris, France. Furthermore with regard to ethical standards for research, the study adhered to the latest release of the revised Declaration of Helsinki in Fortaleza, Brazil (World Medical Association, 2013). The initial page of the online survey contained a consent form and legal information. The two inclusion criteria for respondents were to be employed and to be at least 18-year-old.

## Results

All the data were computed using SPSS (version 23). Following the recommendations of Baron and Kenny (1986), prior to performing a mediation analysis, it was necessary to analyze correlates of adverse working conditions and turnover intentions with meaning of work. In line with this approach, mediation analyses were conducted only among the significant relationships.

### Descriptive Statistics

Means, standard deviations, and correlations of the study variables are presented in **Table 1**. Mean scores of adverse working conditions, and turnover intentions were in the low to moderate range while meaning of work was in the high range.

**Table 1:** Means, standard deviations, and bivariate correlations for all variables.

	1	2	3	4	5	6	7	8
(1) Work pressure	-	0.28^∗∗^	0.26^∗∗^	0.04	0.13^∗∗^	0.08	0.20^∗∗^	-0.03
(2) Lack of resources		-	0.51^∗∗^	0.54^∗∗^	0.31^∗∗^	0.25^∗∗^	0.42^∗∗^	-0.42^∗∗^
(3) Organizational changes			-	0.33^∗∗^	0.25^∗∗^	0.39^∗∗^	0.27^∗∗^	-0.27^∗∗^
(4) Lack of personal development				-	0.36^∗∗^	0.26^∗∗^	0.57^∗∗^	-0.63^∗∗^
(5) Personal reasons					-	0.13^∗^	0.34^∗∗^	-0.28^∗∗^
(6) Public image of the company						-	0.13^∗^	-0.25^∗∗^
(7) Turnover intentions							-	-0.62^∗∗^
(8) Meaning of work								-

*M*	0.46	0.30	0.30	0.28	0.21	0.20	1.96	5.19
*SD*	0.28	0.30	0.28	0.31	0.24	0.35	0.67	0.99


**N* = 336. ^∗∗^*p* < 0.01 (one-tailed), ^∗^*p* < 0.05 (one-tailed).*

As can be seen, adverse working conditions were significantly and positively correlated with turnover intentions, ranging from *r*(336) = 0.13, *p* < 0.05 for public image of the company to *r*(336) = 0.57, *p* < 0.01 for lack of personal development (*mdn* = 0.31). These results suggest that as adverse working conditions increase, turnover intentions also increase. With the exception of work pressure, meaning of work was negatively and greatly associated to adverse working conditions, ranging from *r*(336) = -0.25, *p* < 0.01 for public image of the company to *r*(336) = -0.63, *p* < 0.01 for lack of personal development (*mdn* = -0.28). A negative and significant correlation between meaning of work and turnover intentions was also observed [*r*(336) = -0.62, *p* < 0.01]. That effect is particularly important indicating that as meaning of work increases, turnover intentions decrease. Accordingly, the first hypothesis—that adverse working conditions are positively associated with turnover intentions—was confirmed while the second—that meaning of work is negatively related to adverse working conditions and turnover intentions—was also supported.

### Predicting Turnover Intentions

To examine the predictors of turnover intentions, two hierarchical multiple regression analyses were performed. For the first model, a three step hierarchical multiple regression was conducted, in which demographic data was entered at step 1, adverse working conditions at step 2, and meaning of work at step 3. For the second model, demographic data was also entered in the first step of the equation while meaning of work were included in the second step. Regression results are presented in **Table 2**.

**Table 2:** Hierarchical multiple regression analyses predicting turnover intentions.

	Model 1				Model 2
		
	β	*p*	Δ*R*^2^	β	*p*	Δ*R*^2^
Step 1: Demographic data			0.05			0.05
Gender	0.01	0.79		0.00	0.93	
Age	-0.07	0.15		-0.07	0.18	
Education level	-0.01	0.89		0.01	0.88	
Types of occupation	0.07	0.16		0.03	0.65	
Contract status	0.15	<0.01		0.16	<0.01	
Types of contract	-0.00	0.94		0.03	0.56	
Job tenure	-0.03	0.59		0.02	0.70	
Step 2: Adverse working conditions			0.36			
Work pressure	0.14	<0.01				
Lack of resources	0.06	0.29				
Organizational changes	0.00	0.97				
Lack of personal development	0.24	<0.01				
Personal reasons	0.14	<0.01				
Public image of the company	-0.11	0.02				
Step 3: Meaning			0.10			0.36
Meaning of work	-0.43	<0.01		-0.62	<0.01	
*R*^2^_Total_	0.52			0.41		
*R*^2^_Adjust_	0.49			0.40		


For Model 1, after entering gender, age, education level (i.e., graduate degree level vs. non-graduate degree level), types of occupation (i.e., administrative or managerial positions vs. non-administrative or managerial positions), contract status (i.e., permanent position vs. non-permanent position), types of contract (i.e., full-time position vs. non-full-time position), and job tenure, adverse working conditions uniquely accounted for a significant 36% variance in turnover intentions [*ΔR*^2^ = 0.36, *F*(13,314) = 16.95, *p* < 0.01]. Meaning of work added an incremental and significant 10% in turnover intentions [*ΔR*^2^ = 0.10, *F*(14,313) = 23.73, *p* < 0.01]. More specifically, for the final step, the standardized regression coefficients were significant for contract status (β = 0.15, *p* < 0.01), work pressure (β = 0.14, *p* < 0.01), lack of personal development (β = 0.24, *p* < 0.01), personal reasons (β = 0.14, *p* < 0.01), public image of the company (β = -0.11, *p* < 0.05), and meaning of work (β = -0.43, *p* < 0.01).

For Model 2, after including the same demographic variables, meaning of work contributed significantly to the regression model [*ΔR^2^* = 0.36, *F*(8,319) = 28.18, *p* < 0.01], and accounted for an incremental 36% of turnover intentions. Contract status (β = 0.16, *p* < 0.01) and meaning of work presented a significant standardized regression coefficient (β = -0.62, *p* < 0.01).

### Testing the Mediating Role of Meaning of Work

To examine the role of meaning of work as a mediator of the relationships between adverse working conditions and turnover intentions, the procedure described by Preacher and Hayes (2008) was followed. Thus, based on 5,000 bootstrapped samples using bias-corrected and accelerated 95% confidence intervals, standardized path coefficients and point estimates of indirect effects was calculated for meaning of work. As shown in **Figure 1**, the significant product of the coefficients for the relations between adverse working conditions and the mediator (path *a*) and for the relations between each mediator and turnover intentions (path *b*) was used to determine the significant difference between total effect (path *c*) and indirect effect (path *c’*) as *a* × *b* = *c* -*c’* (MacKinnon et al., 2002). A mediational model for meaning of work was presented in **Table 3**.

**Table 3:** Bootstrapped point estimates and bias corrected and accelerated confidence intervals for indirect effects of adverse working conditions on turnover intentions through meaning of work.

Independent variables	Point estimate	Product of *ab* coefficients			Percent mediated	Bootstrapping 95% CI	
				
		*SE*	*Z*	*p*		Lower	Upper
Work pressure	0.05	0.08	0.62	0.53	-	-0.12	0.22
Lack of resources	0.51	0.07	6.87	<0.01	53.6	0.37	0.68
Organizational changes	0.39	0.08	4.87	<0.01	60.7	0.24	0.57
Lack of personal development	0.60	0.08	7.29	<0.01	48.8	0.44	0.81
Personal reasons	0.45	0.09	5.01	<0.01	47.1	0.29	0.65
Public image of the company	0.31	0.07	4.51	<0.01	-	0.18	0.46


**N* = 336. Percent mediated was calculated following the formula: (1 -*c*’/*c*) × 100.*

Meaning of work was found to be a significant mediator for the relationships of lack of resources (*Z* = 6.87, *p* < 0.01), organizational changes (*Z* = 4.87, *p* < 0.01), lack of personal development (*Z* = 7.29, *p* < 0.01), personal reasons (*Z* = 5.01, *p* < 0.01), and public image of the company (*Z* = 4.51, *p* < 0.01) with turnover intentions. For these four first adverse working conditions factors, mediation effects ranged from 47 to 61% of the overall effect while meaning of work fully mediated the effects of the public image of the company on turnover intentions. Only the effect of work pressure on turnover intentions was not significantly mediated by meaning of work (*Z* = 0.62, *p* = 0.53). These results support the third hypothesis—that effects of adverse working conditions on turnover intentions are partially mediated by meaning of work.

## Discussion

Our study attempted to extend the current literature about the mediating effects of meaning of work, and about the consequences of adverse working conditions on employee outcomes, particularly on turnover intentions. Accordingly, we hypothesized that increased levels of adverse working conditions would lead to increased levels of turnover intentions. As predicted, adverse working conditions were positively and significantly associated with high turnover intentions, showing correlations at different ranges. These results support previous studies that found similar relationships among diverse working groups (Houkes et al., 2001; Huang et al., 2007; Podsakoff et al., 2007; Poilpot-Rocaboy et al., 2011; Burakova et al., 2014). However, Podsakoff et al. (2007) found working conditions related to task accomplishment to be better predictors of turnover intentions than working conditions related to personal development. Such differential effects between these two categories were found among our sample, for lack of resources and personal development were greatly associated with turnover intentions. As postmodernist societies encourage individuals to take full responsibility for their personal development (Guichard et al., 2012), we can argue that lack of personal development within an organizational context is becoming a more and more critical factor for turnover intentions (Freund, 2005).

Secondly, we postulated that high levels of meaning of work would be associated with low adverse working conditions and turnover intentions. All these predictions were confirmed. A previous study also found a strong correlation between meaning of work and turnover intentions (Steger et al., 2012).

Finally, our study explored the role of meaning of work as a mediator of the relationships between adverse working conditions and turnover intentions. The mediating effect of meaning of work was clearly demonstrated for four adverse working conditions (i.e., lack of resources, organizational changes, lack of personal development, and personal reasons). These findings are convergent with the JCM postulating the job characteristics influence critical psychological states, with in return have significant and various impacts on employee’s work outcomes (Hackman and Oldham, 1976; Humphrey et al., 2007). It supports meaning of work as a significant psychological resource for mediating the negative effects of working conditions on turnover intentions, in addition to other previously identified psychological resources (Aryee and Chay, 2001; Loi et al., 2006; Humphrey et al., 2007; Podsakoff et al., 2007; Kim and Stoner, 2008; Vandenberghe and Tremblay, 2008; Collins, 2010). In their meta-analysis, Humphrey et al. (2007) underlined the importance of meaningful work as the most important psychological resource to prevent negative employee’s work outcomes.

### Limitations

The findings of this study should be considered in the light of several limitations. Adverse working conditions were measured by calculating indexes derived from dichotomous observed variables. Nearly all reliability coefficients of adverse working condition factors were satisfied, but some factors presented tolerable or even unacceptable values. Indeed, despite the relevance of job insecurity (Sverke et al., 2002) and hostile work climate (Carr et al., 2003) as decidedly adverse working conditions, these factors were excluded from the analyses due to their limited internal consistencies. Further analyses revealed that few respondents concurrently encountered multiple situations related to each factor. For instance, among our sample, only 10% of participants reported experiencing “assault at work” (item 16 in the adverse working conditions questionnaire), which was significantly marginal in relation to the majority of respondents [χ^2^(1) = 213.76, *p* < 0.01]. However, we should consider that every employee experiences each situation at work on a certain level. Thus, assessing working conditions or work events by use of a continuum rather than dichotomous scoring may enhance reliabilities (for examples, see Cohen et al., 1993; Van Os et al., 2001; Stöber et al., 2002). Consequently, continuous variables may also provide opportunities to further examine the implications of rare adverse working conditions on employee outcomes.

In addition, only turnover intentions were investigated in our study. Because turnover intentions are considered as antecedents of turnover (Podsakoff et al., 2007), the mediating effect of meaning of work between working conditions and actual turnover could differ. Indeed, turnover intentions more describe a psychological state whereas turnover represents an effective decision and behavior taken by employees. Accordingly, longitudinal research designs may provide further information about the underlying process. Overall, understanding turnover intentions may lead to decreased turnover.

Another concern is the nature of our sample, which mainly consisted of respondents who had attended or completed a graduate degree program and who held administrative or managerial positions. This composition may potentially explain the high level of meaning of work that was observed in our study. Indeed, as demonstrated by Allan et al. (2014), employees from higher social classes tend to experience higher meaning of work than employees in lower social classes. Nevertheless, we conducted additional analyses which revealed that demographic data accounted for a negligible amount of variance in meaning of work and that none of these variables significantly predicted turnover intentions. In sum, future studies among large representative working samples are needed to better understand the implications of both job and personal characteristics. Special attention is also required to include individuals living in extremely precarious conditions, who may not be readily accessible for researchers (Rhodes et al., 2003), but who may represent a promising area for understanding the relationships between adverse working conditions and meaning of work. An intriguing and paradoxical example was provided by Arvidsson et al. (2010) among Milan fashion industry employees who experienced their work as meaningful despite poor working conditions.

### Perspectives

In the range of the JCM’s perspectives (Hackman and Oldham, 1976), our study makes a significant contribution to understanding the role of meaning of work as a significant psychological resource for reducing turnover intentions while employees are facing adverse working conditions. Future research should further explore its implications on other negative employee outcomes (e.g., absenteeism, burnout, and turnover), as well as on positive employee outcomes (e.g., job satisfaction, motivation, and organizational commitment). Despite the growth of substantial literature about the benefits of meaning of work for both workers and organizations (see Rosso et al., 2010; Steger et al., 2012), the mediating role of meaning of work—in the negative context of poor working conditions and psychosocial risks for employee outcomes—remains unclear. This research topic seems promising since ongoing changes in work organization, marked by constant flux, job insecurity, and flexibility, increase the importance and relevance of considering meaning of work as a critical psychological resource among workers (Hartung and Taber, 2013; Bernaud et al., 2015). Finally, in addition to promoting decent working conditions, organizations should encourage programs and interventions that help employees to develop positive psychological resources, including meaning of work.

## Author Contributions

All the authors (CA-N, LS, LL, AF, and J-LB) substantially contributed to the conception and the design of the work. CA-N, LS, LL, and J-LB participated to the acquisition of data. The two first authors (CA-N, LS) analyzed and interpreted the data. The first author (CA-N) prepared the draft and the contributing authors (LS, LL, AF, and J-LB) reviewed it critically and gave important intellectual content. All the authors (CA-N, LS, LL, AF, and J-LB) worked for the final approval of the version to be published. All the authors (CA-N, LS, LL, AF, and J-LB) are accountable for all the aspects of the work in ensuring that questions related to the accuracy or integrity of any part of the work are appropriately investigated and resolved.

## Conflict of Interest Statement

The authors declare that the research was conducted in the absence of any commercial or financial relationships that could be construed as a potential conflict of interest.
